# Three-Dimensional quantitative analysis of the Peri-Enhancing zone reveals ADC and CBV signatures of glioblastoma recurrence

**DOI:** 10.1016/j.nicl.2026.103977

**Published:** 2026-03-04

**Authors:** Gergely Bertalan, Nicolin Hainc, Gaetan Paignon, Ramona A. Todea, Andrea Bink, Tobias Weiss, Michael Weller, Carlo Serra, Zsolt Kulcsar

**Affiliations:** a Department of Neuroradiology, University Hospital Zurich, Switzerland; b Institute of Neurology, University Hospital Zurich, Switzerland; c Department of Neurosurgery, University Hospital Zürich, Switzerland

**Keywords:** Glioblastoma, Infiltration zone, Magnetic resonance imaging, Quantitative imaging, Volumetric evaluation

## Abstract

**Purpose:**

Accurate detection of glioblastoma infiltration and early *peri*-enhancing changes that predict recurrence remains challenging. We present a three-dimensional (3D) quantitative framework to characterize the *peri*-enhancing zone (PEZ) on baseline and follow-up MRI and examine whether baseline ADC and CBV differentiate regions that subsequently recur from those that remain stable.

**Methods:**

We retrospectively analyzed patients with IDH wild-type glioblastoma who developed local recurrence within 12 months post-resection. The contrast-enhancing tumor core including the necrotic region, and surrounding FLAIR hyperintensity were segmented in 3D on diagnostic MRI. Local recurrence was identified on follow-up MRI as abnormal T1 enhancement contiguous with the initial T1-enhancing core and mapped onto baseline images. The PEZ (up to 5 voxels surrounding the T1-enhancing core) was divided by FLAIR signal into hyperintense (PEZ_FLAIR+_) and non-hyperintense (PEZ_FLAIR-_) subvolumes. Within these subvolumes, voxels overlapping with future T1 abnormalities were classified as recurrence-prone tissue (PEZ_T1+_), while voxels without subsequent enhancement were designated as normal remaining tissue (PEZ_T1-_), yielding four analytical subvolumes for quantitative region-of-interest–based analysis. Mean ADC and CBV were calculated per subvolume and compared using Wilcoxon signed-rank tests and Pearson correlations.

**Results:**

Of 101 eligible patients, 59 developed local recurrence and 46 were included in the final analysis. The mean PEZ_T1+_ volume was 6312 ± 4131 mm^3^, with approximately 30% located in (PEZ_FLAIR-_). Within PEZ_FLAIR+_, PEZ_T1+_ regions showed 8.2% lower ADC (p≈0.0007) and 13.5% higher CBV (p≈0.006) than PEZ_T1-_, with significant ADC–CBV correlation (p < 0.0005). No significant differences or correlations were observed in PEZ_FLAIR-_.

**Conclusions:**

3D volumetric analysis of the PEZ reveals distinct ADC and CBV signatures in regions predisposed to recurrence, which are not apparent on conventional MRI. Differences were confined to FLAIR-hyperintense regions, highlighting the need for novel imaging strategies to detect at-risk tissue in FLAIR-normal PEZ. Importantly, we present a new 3D evaluation approach linking baseline and follow-up MRI that can be applied to investigate other clinical and research questions in brain tumor imaging.

## 1 Introduction

Glioblastoma is the most common primary brain tumor in adults, with a median overall survival of only 15 months despite multimodal therapy (Louis, 2021). Tumor recurrence is nearly universal, typically occurring within 2–3 cm of the initial lesion or resection cavity, even after gross total resection and standard adjuvant radiochemotherapy (Brandes, 2009, Chamberlain, 2011, Nestler, 2015). Extent of resection is a major prognostic factor (Stupp, 2005, Vaz-Salgado, 2023, Weller, 2021), motivating interest in supramarginal resection strategies that extend beyond contrast-enhancing tumor margins to include adjacent regions at high risk of microscopic infiltration (Guerrini et al., 2022, Wach et al., 2023). It is believed that a better preoperative estimation of tumor infiltration with imaging could significantly optimize target volumes for supramarginal resection and postoperative radiation therapy and minimize neurological risk by identifying *peri*-enhancing tissue areas with high tumor infiltration and high likelihood of recurrence (Guerrini et al., 2022, Wach et al., 2023). However, it remains unclear whether MRI can accurately detect tumor infiltration and identify areas that correspond to regions at high risk of recurrence beyond the visible T1-weighted contrast-enhancing core (Niyazi, 2023).

Glioblastoma typically presents as a heterogeneous contrast-enhancing mass surrounded by T2/FLAIR hyperintensity, reflecting a mixture of edema and infiltrating tumor (Martin-Noguerol et al., 2021). Although definitions vary, the tissue surrounding the contrast-enhancing core can be conceptually subdivided into concentric zones: the *peri*-enhancing zone (PEZ, up to 5 mm), the near zone (5–10 mm), and the far zone (10–15 mm) (Lemée et al., 2015). Unlike the core, which is generally removed during surgery, these surrounding regions are largely preserved and accessible for longitudinal imaging. Among them, the PEZ is particularly important for recurrence, as it lies immediately adjacent to the contrast-enhancing core and represents the invasion front, where most local relapses arise (Lemée et al., 2015, Giambra, 2023). Histopathologically, the PEZ comprises a dynamic mixture of infiltrating tumor cells, extracellular matrix alterations, and edema, exhibiting more pronounced tissue changes than the near and far zones (Lemée et al., 2015, Giambra, 2023). This biological gradient of tumor infiltration positions the PEZ as a critical region for early detection of residual tumor and progression, making it a prime target for imaging studies aimed at characterizing tumor invasion beyond the contrast-enhancing core.

Quantitative MRI (qMRI) provides numeric biomarkers that are more sensitive to subtle tissue changes than conventional MRI. Diffusion-weighted imaging (DWI) and dynamic susceptibility contrast (DSC) MRI are widely used in clinical glioblastoma assessment (Martin-Noguerol et al., 2021, Blystad, 2017). DWI-derived apparent diffusion coefficient (ADC) maps quantify voxel-wise water diffusivity, with low ADC in the contrast-enhancing core reflecting high cellularity and higher ADC in the surrounding PEZ reflecting edema (Baehring and Fulbright, 2012, Gaddamanugu, 2022). DSC MRI provides perfusion metrics such as cerebral blood volume (CBV), informing tumor vascularity, angiogenesis, and potentially aggressiveness (Essig, 2013, Anzalone, 2018). Both ADC and CBV have demonstrated utility in surgical planning, tumor grading, and differentiating pseudoprogression from true progression (Essig, 2013, Anzalone, 2018).

Despite these capabilities, local recurrence remains relatively unpredictable. Biological alterations that drive recurrence, including increased tumor cell density and extracellular matrix degradation, are often present in the PEZ at diagnosis (Lemée et al., 2015, Giambra, 2023). Glioblastoma growth generally follows white matter tracts, ventricles, or cerebrospinal fluid pathways (Fares, 2025, Tsuchiya, 2024), yet infiltration in the PEZ is highly heterogeneous, with some subregions prone to progression while others remain spared (Lemée et al., 2015, Giambra, 2023). Current clinical criteria, including RANO (Wen, 2023), primarily focus on changes in the contrast-enhancing lesion, with non-enhancing FLAIR or T2 abnormalities considered supportive. Consequently, most imaging studies emphasize the contrast enhancing tumor core, and three-dimensional quantitative analyses of the PEZ remain scarce. Prior investigations of the PEZ have largely relied on two-dimensional, region-of-interest–based assessments or limited slices (Martin-Noguerol et al., 2021, Michalska-Foryszewska, 2024), highlighting the need for robust, volumetric methods to evaluate the PEZ in a clinically applicable manner.

While several studies have attempted to predict glioblastoma recurrence using conventional or deep learning approaches (Hu, 2023, Li, 2025, Wang, 2021, Shim, 2021), published methods often perform poorly in practice, particularly those relying solely on anatomical MRI sequences such as T1, T1c, T2, or FLAIR. A fundamental question remains unresolved: does diagnostic MRI contain detectable signals that differentiate tissue destined to recur from tissue that remains stable? Quantitative MRI metrics, such as ADC and CBV, are particularly suited for this purpose because they are more sensitive to microstructural and vascular tissue changes than standard anatomical MRI. Although prior studies have employed 3D or distance‑resolved quantitative MRI analyses around enhancing glioblastoma regions (Blystad, 2017, Lemercier et al., 2014, Bertalan, 2025); to our knowledge, comprehensive three‑dimensional evaluation of the preoperative PEZ using both ADC and CBV co‑registered to subsequent recurrence patterns remains limited.

The primary research question of this study is whether diagnostic qMRI metrics (ADC and CBV) differ between *peri*-enhancing tissue that subsequently develops recurrence and tissue that remains relatively stable. To address this question, we introduce a clinically motivated 3D imaging approach to evaluate the PEZ across diagnostic and follow-up MRI. Our primary objectives are threefold: 1) to identify imaging signatures within PEZ subregions associated with subsequent recurrence; 2) to establish a robust image preprocessing pipeline for systematic evaluation of baseline PEZ characteristics; and 3) to highlight the radiological relevance of the PEZ in glioblastoma recurrence, thereby supporting future investigations of tumor infiltration and progression.

## 2 Methods

### 2.1 Patients

**Fig. 1:**
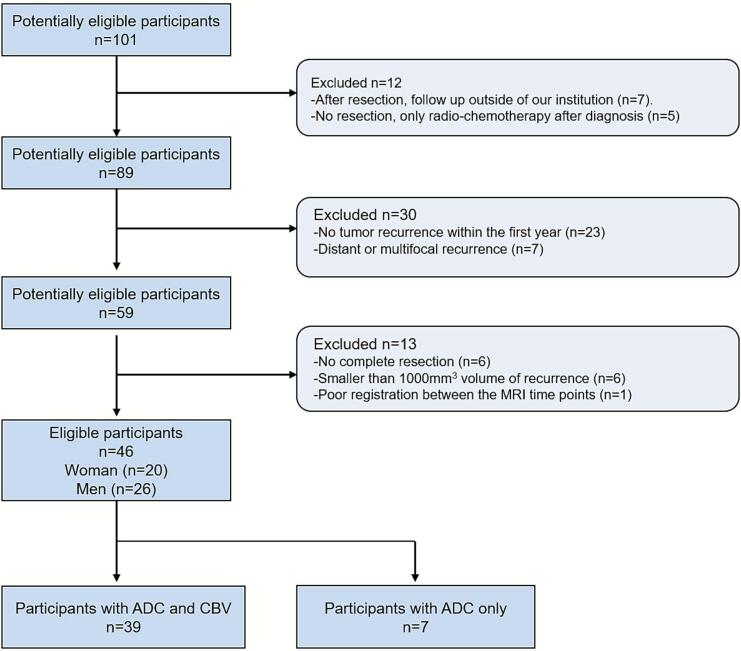
Patient-selection protocol and criteria for inclusion and exclusion.

We performed a retrospective analysis of 101 patients with isocitrate dehydrogenase (IDH) wild-type glioblastoma diagnosed between 2018 and 2022, as previously reported (*blinded for review*). Patient selection and inclusion/exclusion criteria are summarized in Fig. 1. Inclusion criteria were: (1) pre- and postoperative follow-up MRI performed on the same Siemens scanner (Erlangen, Germany) using a standard brain tumor protocol; (2) availability of T1, T1-contrast enhanced (T1c), T2, FLAIR, diffusion-weighted imaging (DWI), and dynamic susceptibility contrast (DSC) perfusion MRI; (3) visually apparent preoperative T1-enhancing tumor ≥ 1000 mm^3^; (4) resection leaving ≤ 500 mm^3^ residual T1-enhancing tumor; (5) visually apparent T1-enhancing recurrence within 12 months post-diagnosis ≥ 1000 mm^3^; (6) confirmed tumor recurrence excluding therapy-related changes based on MRI, positron emission tomography, and histology; and (7) recurrence contiguous with the original contrast-enhancing core and/or the resection cavity. Integrated molecular and histopathological diagnosis confirmed IDH wild-type glioblastoma in all patients. Resection quality and residual T1-enhancing tumor volume were assessed via intraoperative or postoperative MRI within 48 h after surgery. All patients underwent tumor resection followed by standard concomitant radiochemotherapy. The study was approved by the regional ethics board (*blinded for review*, ethical application number: *blinded for review*) in accordance with the Declaration of Helsinki.

### 2.2 Imaging

MRI was performed on a 3 Tesla scanner (Skyra; Siemens, Erlangen, Germany) with participants in the supine position using a standard clinical brain tumor protocol. The protocol included pre-contrast 3D T1, 2D T2, 2D DWI with ADC maps, 2D DSC, 3D FLAIR, and 3D T1c sequences.

Two imaging time points were analyzed for recurrence. The first (MRI_baseline_) corresponded to the preoperative diagnostic MRI acquired within five days before resection; all tumors were treatment-naïve at this time. The second (MRI_reccurrence_) corresponded to the first follow-up MRI within 12 months after MRI_baseline_ in which tumor progression was identified. Conventional sequences (T1, T1c, T2, FLAIR) were acquired using standard parameters (Sanvito et al., 2023).

DSC was acquired using a 2D spin-echo echo-planar imaging sequence with a field of view of 220 × 220 mm, 24 slices, 128 × 128 voxel matrix, in-plane voxel size 1.7 × 1.7 mm, slice thickness 4 mm, 60 dynamic measurements with 1.8 s temporal resolution, TE = 29 ms, TR = 1630 ms, and GRAPPA acceleration factor of 2. Total acquisition time was 1:51 min.

Semi-quantitative cerebral blood volume (CBV) maps were reconstructed using a standard procedure (Zhang, 2017, Bjornerud and Emblem, 2010) implemented in Python 3.16 (Python Software Foundation (Foundation, P.S. Python Software Foundation). Time-resolved signal intensity curves were first converted voxel-wise into contrast agent concentration curves using:

$$C\left(t\right)\approx -\frac{1}{\mathrm{TE}}ln\left(\frac{S(t)}{S_{0}}\right)$$

where *S(t)* is the DSC signal intensity at time *t, S_0_* is the baseline signal before contrast injection and TE is the echo time. Relative cerebral blood volume (rCBV) was then estimated using a slice-wise arterial input function (AIF) approach (Bjornerud and Emblem, 2010). For each transverse slice, voxel-wise concentration–time curves C(x,y,t) were inspected to identify candidate vascular voxels based on peak signal intensity:

$$peaks\left(x,y\right)=\max_{t}C\left(x,y,t\right);candidatemask=\left\{\left(x,y\right):peaks\left(x,y\right)\geq P_{\mathrm{top}}\right\}$$

where *P_top_* denotes the 98th percentile of voxel peaks within the slice. A minimum of ten voxels was enforced to ensure robust estimation. The slice-wise AIF was defined as the median across candidate voxel curves:

$$AIF\left(t\right)={\mathrm{median}}_{\left(x,y\right)\in candidatemask}C\left(x,y,t\right)$$

To reduce noise, the AIF was smoothed using a Gaussian filter (σ = 1 time point). Voxel-wise rCBV was then computed as the ratio of the integral of the concentration–time curve to that of the slice-wise AIF:

$$rCBV\left(x,y\right)=\frac{\int_{0}^{T}C\left(x,y,t\right)dt}{\int_{0}^{T}AIF\left(t\right)dt}$$

where integration was performed using the trapezoidal rule and *T* represents the total acquisition duration.

DWI was acquired using a 2D spin-echo echo-planar imaging sequence with segmented k-space readout (RESOLVE, Siemens) with a field of view of 220 × 220 mm, 34 slices, 190 × 190 voxel matrix, in-plane voxel size 1.2 × 1.2 mm, slice thickness 3 mm, two b-values (0 and 1000 s/mm^2^), TE1 = 71 ms, TE2 = 117 ms, TR = 7220 ms, GRAPPA acceleration factor of 2, and 7 read-out segments. Total acquisition time was 4:29 min. Quantitative ADC maps were automatically generated by the scanner based on the acquired b-values.

### 2.3 Image processing

**Fig. 2:**
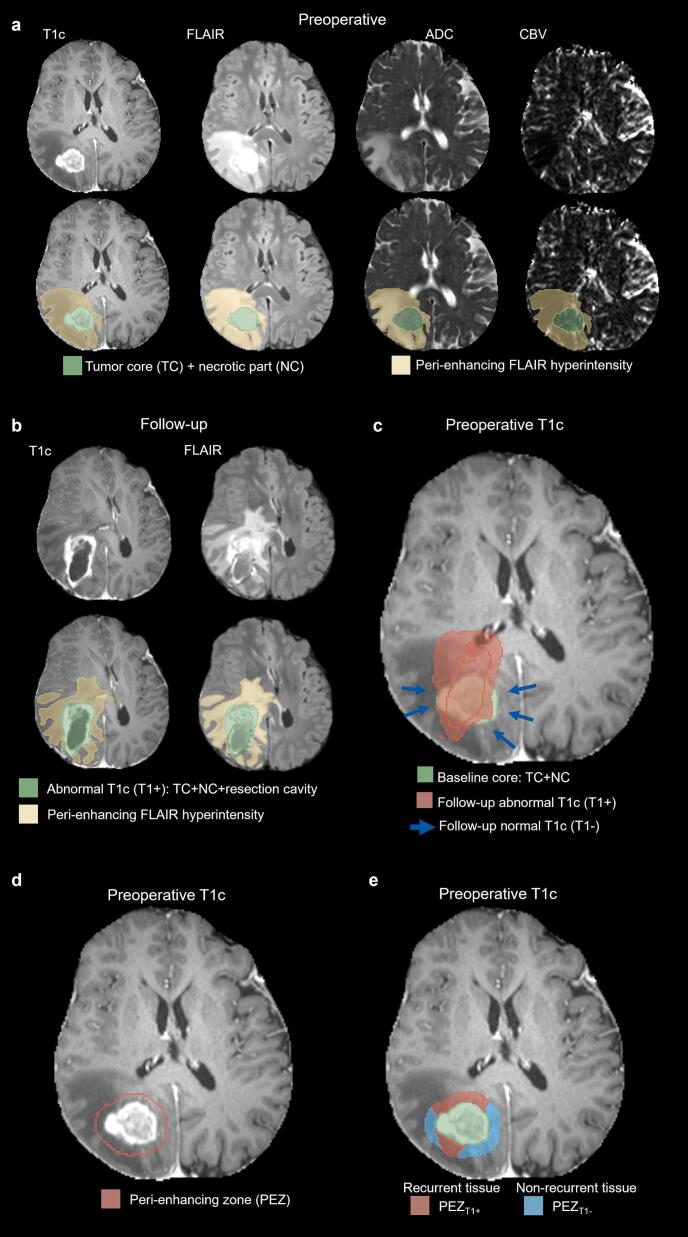
Segmentation of the *peri*-enhancing zone in a 65-year-old female with glioblastoma. (a) Preoperative T1-contrast-enhanced (T1c), FLAIR, ADC, and CBV images. The gross tumor volume, including the contrast-enhancing tumor and necrotic core, is shown in green. (b) Follow-up T1c and FLAIR images 8 months post-resection demonstrating clinically confirmed tumor recurrence. The abnormal T1c segment (T1 + ) is highlighted in green. After this follow-up MRI, the patient received a second surgery. (c) Overlay of T1+ (red) on the preoperative T1c. Blue arrows indicate areas around the baseline tumor core without subsequent T1c enhancement (T1-). (d) The *peri*-enhancing zone (PEZ) defined as a 5-voxel-wide margin surrounding the 3D contours of the gross tumor. (e) Subdivision of the preoperative PEZ into regions of future normal tissue (PEZ_T1-_) and future abnormal tissue (PEZ_T1+_) based on follow-up MRI. (For interpretation of the references to colour in this figure legend, the reader is referred to the web version of this article.)

**Fig. 3:**
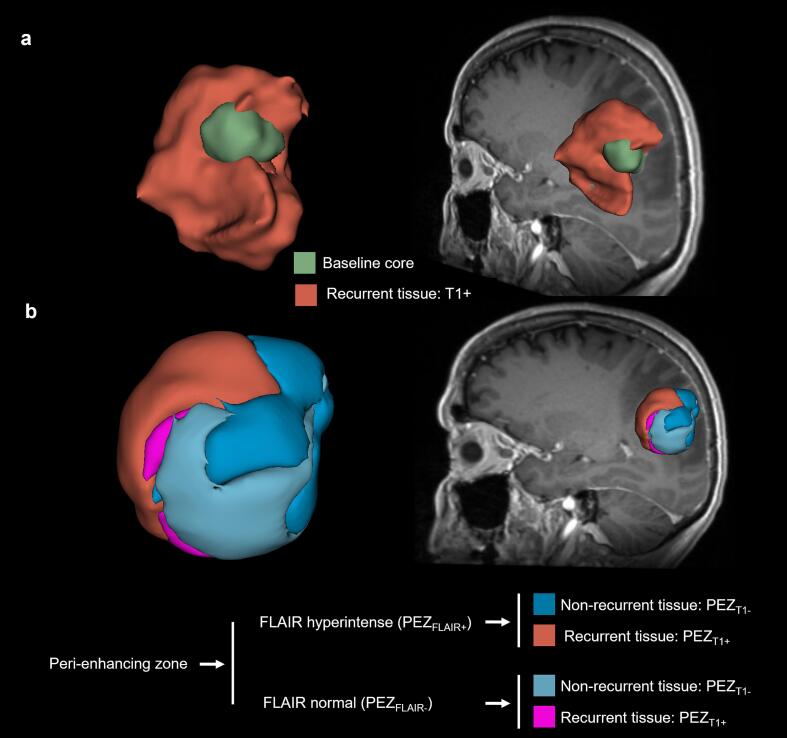
Three-dimensional segmentation of the *peri*-enhancing zone (PEZ) for the patient shown in Fig. 2. (a) Overlay of the total follow-up T1 + volume (red) on the preoperative gross tumor (green). (b) Regions of future normal tissue (PEZ_T1-_) and future abnormal tissue (PEZ_T1+_) of the PEZ were further divided based on preoperative FLAIR hyperintensity. (For interpretation of the references to colour in this figure legend, the reader is referred to the web version of this article.)

**Fig. 4:**
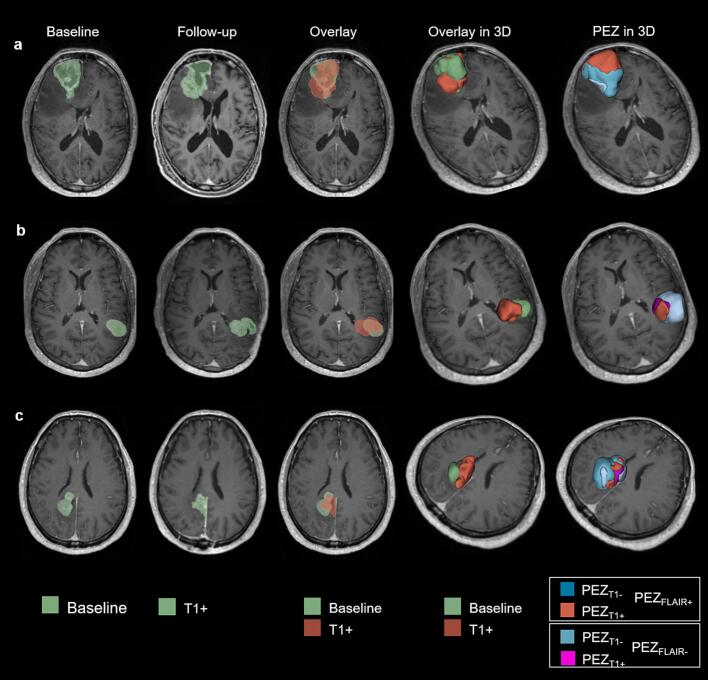
Examples of follow-up MRI registration to baseline scans and corresponding 3D segmentations of the *peri*-enhancing zone (PEZ) in three patients with glioblastoma. (a) 69-year-old male with recurrence 6.5 months after diagnosis; (b) 69-year-old female with recurrence 4.5 months after diagnosis; (c) 55-year-old male with recurrence 7.5 months after diagnosis. All patients underwent a second resection following follow-up MRI.

Postprocessing and statistical analyses were performed using Python (Foundation, P.S. Python Software Foundation) (Python Software Foundation, https://www.python.org) and Matlab 2022 (The MathWorks, Natick, MA, USA). The processing pipeline comprised seven sequential steps: (1) image resampling and within-time-point registration; (2) skull stripping; (3) registration of MRI_reccurrence_ to MRI_baseline_; (4) tumor segmentation at both time points; (5) generation of the PEZ surrounding the T1-enhancing tumor on MRI_baseline_; (6) overlay of recurrent tumor segments on MRI_baseline_ and subdivision of the PEZ zone accordingly; and (7) statistical analysis of ADC and CBV within the resulting 3D volumes. The postprocessing workflow and representative volumes are illustrated in Fig. 2, Fig. 3, Fig. 4.

Within each imaging time point, all images were resampled to a uniform 256 × 256 × 220 matrix with 1 mm isotropic resolution. Co-registration to the T1c was performed using DIPY (Garyfallidis, 2014) (https://www.dipy.org), which provides an optimized, built-in registration pipeline. In this framework, the user specifies a template and a moving MRI volume and can select the registration method (rigid, affine, or sequential rigid followed by affine). This registration procedure ensures spatial alignment of all MRI sequences within each time point before further preprocessing.

Skull stripping is a necessary prerequisite for automated tumor segmentation, as non-brain structures can have similar MRI signal characteristics (T1, T2, FLAIR) to glioblastoma and could otherwise be incorrectly identified as tumor. Skull stripping was performed using a self-trained U-Net (Ronneberger et al., 2015) (*blinded for review*). This step generates a mask that removes all structures outside the brain, such as the skull, scalp, and eyes, while retaining the entire intracranial contents, including brain tissue and all internal anatomical structures. The resulting brain mask was visually inspected and, if necessary, manually corrected by expert readers to ensure accurate extraction of the intracranial contents. Tumor segmentation was then applied exclusively to the skull-stripped images.

The T1 of MRI_reccurrence_ was co-registered to the T1 of MRI_baseline_ using a three-step semi-automatic procedure with DIPY (Garyfallidis, 2014). First, rigid registration was applied to the skull-stripped MRI volumes, aligning the follow-up skull to the baseline skull to generate an initial approximation. Second, this transformation was refined by performing rigid followed by affine registration on the full MRI volumes (skull + brain). Third, if minor residual misalignments remained after the automated steps—typically due to post-surgical changes or subtle brain deformations—manual refinement was performed using the 3D Slicer (Fedorov, 2012) registration module and linear adjustments, including small translations and rotations. This step was required only in a small fraction of the cohort. During manual refinement, the alignment of the MRI volume was adjusted to maximize correspondence of key anatomical structures, including ventricles and tumor boundaries. The final transformation matrix was then applied to all MRI volumes in MRI_recurrence_. Registration quality was systematically evaluated by expert review to ensure anatomically accurate alignment.

In both MRI_baseline_ and MRI_reccurrence_, glioblastomas were automatically segmented into contrast enhanced tumor core including the T1-enhanced tumor and corresponding necrotic part, and peritumoral FLAIR hyperintensity using a self-trained U-Net model leveraging standard MRI contrasts (T1, T1c, T2, and FLAIR), as described in our previous work (*blinded for review*). The same segmentation pipeline and expert-review process were applied consistently to both baseline and follow-up MRI to ensure comparability of ROIs across time points. Automatic tumor segmentation was systematically reviewed and, where necessary, manually corrected in 3D Slicer (Fedorov, 2012) by consensus between (blinded for review) and (blinded for review). The deep learning–based segmentation was used solely as an anatomical preprocessing step to define regions of interest (ROI) for all subsequent quantitative analyses, which were performed exclusively on these expert-validated ROIs. Conceptually, the same analysis could have been performed using alternative established brain tumor segmentation tools; the use of an in-house U-Net reflects a practical implementation choice rather than a methodological dependency of the study.

For MRI_baseline_, the gross total tumor was defined as the T1-enhanced tumor core together with the corresponding necrotic area encompassed by T1 hyperintensity (Fig. 2a). In MRI_reccurrence_, the abnormal T1c volume was defined as the union of the T1-enhanced volume and the associated necrotic region (Fig. 2b). Because the resection cavity often persists for months or permanently, may be filled with cerebrospinal fluid, and exhibits T2 and FLAIR characteristics similar to necrosis, it was often identified by the segmentation model as necrotic tissue; therefore, abnormal T1c volume also included the residual resection cavity encompassed by T1 enhancement (Fig. 2, Fig. 3).

All subsequent analyses focus on the treatment-naïve PEZ on MRI_baseline_. Owing to its small size, anatomical constraint, and proximity to the tumor core, alignment between baseline and follow-up MRI can be achieved with relatively high local accuracy within the PEZ, even when larger-scale brain distortions are present. The PEZ on MRI_baseline_ was defined as a 5-voxel rim surrounding the contrast-enhancing tumor core, generated using the Visualization Toolkit (Schroeder and M.K., Lorensen B. , 2006) (VTK, https://www.vtk.org), as illustrated in Fig. 2d. To account for known heterogeneity, the PEZ was subdivided according to FLAIR signal into hyperintense (PEZ_FLAIR+_) and non-hyperintense (PEZ_FLAIR-_) regions. Postoperative follow-up scans were used to delineate abnormal T1 contrast enhancement corresponding to local recurrence, which was then co-registered and overlaid onto the MRI_baseline_. Within the PEZ, voxels overlapping with future T1 abnormalities were classified as recurrence-prone tissue (PEZ_T1+_), while voxels without subsequent enhancement were designated as normal remaining tissue (PEZ_T1-_) (Fig. 2c, e). This approach generated four analytical subvolumes for quantitative region-of-interest-based analysis: PEZ_T1+_ and PEZ_T1-_ – within both PEZ_FLAIR+_ and PEZ_FLAIR-_ compartments (Fig. 3).

### 2.4 Statistical analysis

All images were resampled to an isotropic resolution of 1 mm, and tumor segment volumes were obtained by voxel counting (voxel size = 1 mm^3^). Segmented regions were overlaid onto the corresponding ADC and CBV maps. To avoid contamination from non-brain tissue, voxels within PEZ that overlapped with ventricles or cortical sulci were excluded using an ADC-based threshold mask. ADC thresholds were manually adjusted for each patient under expert supervision following a standardized procedure based on histogram inspection. This approach accounted for inter-subject variability in cerebrospinal fluid and ventricular signal, ensuring accurate exclusion of fluid-filled compartments while preserving brain parenchyma, including tumor-infiltrated tissue.

Group-level comparisons were performed according to standard neuroradiological practice, as follows. Voxel-wise ADC maps were obtained directly from the scanner, while rCBV maps were generated as described above. Each patient contributed four ROIs that were overlaid onto the ADC and rCBV maps: 1) PEZ_T1+_ within PEZ_FLAIR+_, 2) PEZ_T1-_ within PEZ_FLAIR+_, 3) PEZ_T1+_ within PEZ_FLAIR-_, and 4) PEZ_T1-_ within PEZ_FLAIR-_. For each ROI, the mean and standard deviation (SD) of ADC and CBV values were calculated yielding four mean ± SD values per patient. For each ROI, mean ADC and rCBV values were derived using three complementary approaches: (i) all voxels within the segment, (ii) voxels after excluding the lowest 20th percentile (yielding ADC_high_ and rCBV_high_), and (iii) voxels after excluding the highest 20th percentile (yielding ADC_low_ and rCBV_low_). Group-level comparisons of ADC and rCBV between PEZ_T1+_ and PEZ_T1-_ regions were performed separately within PEZ_FLAIR+_ and PEZ_FLAIR-_ subvolumes using the two-sided Wilcoxon–Mann–Whitney test. Associations between ADC and rCBV were evaluated using Pearson’s correlation coefficient. Statistical significance was defined as p < 0.05. The discriminative ability of ADC and rCBV was assessed descriptively using receiver operating characteristic (ROC) analysis and simple threshold-based classification. The area under the ROC curve (AUC) was calculated to assess the ability of each parameter to discriminate between PEZ_T1+_ and PEZ_T1-_ regions. Optimal thresholds were determined by maximizing Youden’s index.

To reduce inter-subject variability and enable consistent group-level comparisons, ADC and rCBV maps were normalized using a histogram-based scaling procedure. For each patient, voxel-wise distributions of ADC and rCBV values across the whole brain were used to construct cumulative histograms, which were then linearly scaled to a cohort-level mean reference range. This approach preserves relative contrast between tissue classes while minimizing global intensity differences introduced by acquisition variability. Importantly, absolute ADC and rCBV values were evaluated directly, without the use of reference regions such as normal-appearing white matter, which are commonly employed in clinical studies. A detailed rationale for this normalization strategy is provided in the Discussion.

## 3 Results

### 3.1 Cohort characteristics

Of 101 potentially eligible patients, 46 met inclusion criteria and were included in the analysis (Table 1, Fig. 1). Exclusions were primarily due to absence of recurrence within 12 months post-resection (n = 23), multifocal or distant recurrence (n = 7), external follow-up (n = 7), non-standard treatment (n = 5), partial resection (n = 6), small recurrence volumes (n = 6), or poor registration quality (n = 1).

**Table 1:** Cohort characteristics.

**Characteristic**	**n = 46**
Age (years)	58 ± 11
Sex (M/F)	20 /26
IDH status	All wild-type
Recurrence	All cases within 12 months post-resection
Time to recurrence (months)	5.76 ± 2.5

### 3.2 Volumetric characteristics of recurrent and non-recurrent tissue

**Fig. 5:**
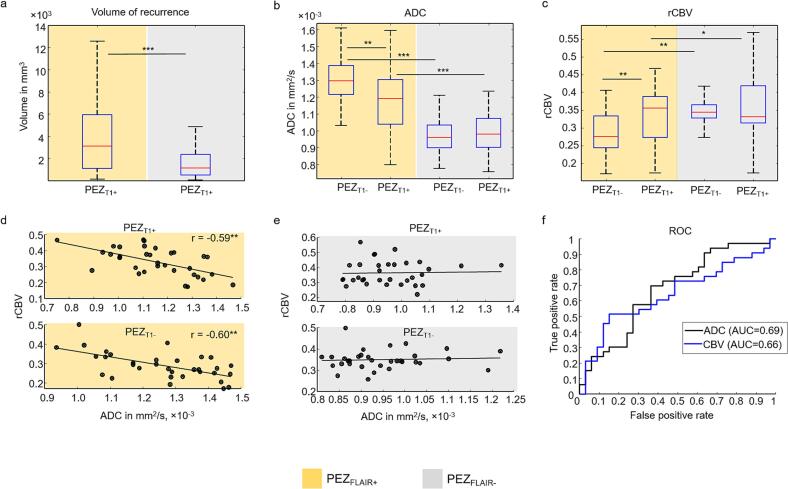
Volumetric, ADC, and CBV analysis of the *peri*-enhancing zone (PEZ). (a) Volume of postoperative T1 contrast enhancement within the preoperative PEZ (PEZ_T1+_). Group-mean PEZ_T1+_ volume was significantly larger in FLAIR-hyperintense PEZ (PEZ_FLAIR+_) than in FLAIR-normal PEZ (PEZ_FLAIR–_), with approximately one-third of PEZ_T1+_ volume arising in PEZ_FLAIR–_. (b) ADC values in the PEZ. PEZ_FLAIR+_ regions exhibited higher ADC than PEZ_FLAIR-_. Within PEZ_FLAIR+_, recurrent regions (PEZ_T1+_) showed significantly lower ADC than non-recurrent regions (PEZ_T1-_), whereas no differences were observed in PEZ_FLAIR–_. (c) rCBV values in the PEZ. rCBV was significantly lower in PEZ_FLAIR+_ than in PEZ_FLAIR-_. Within PEZ_FLAIR+_, PEZ_T1+_ exhibited significantly higher rCBV than PEZ_T1-_, whereas no differences were observed in PEZ_FLAIR-_. (d) Correlation between ADC and rCBV in PEZ_T1+_ and PEZ_T1-_ within PEZ_FLAIR+_. In both regions, higher ADC was significantly associated with lower rCBV. (e) The same analysis as in (d) for PEZ_FLAIR-_. No significant correlations were observed. (f) ROC–AUC analysis of group-mean ADC and rCBV values. ADC yielded an AUC of 0.69 with an optimal threshold of 1227 × 10^–6^ mm^2^/s, while rCBV achieved an AUC of 0.66 with an optimal threshold of 0.355. Significance levels from Wilcoxon signed-rank tests and Pearson’s correlation are indicated as follows: *p < 0.05, **p < 0.005, ***p < 0.0005.

Fig. 2e provides a visual definition of recurrent and non-recurrent tissue within the *peri*-enhancing zone (PEZ_T1+_ vs. PEZ_T1−_). Table 2 summarizes the measured volumes for the analyzed subregions. Although recurrence is typically expected in FLAIR-hyperintense regions (PEZ_FLAIR+_), approximately one-third of the recurrent tissue within the PEZ was located in FLAIR-normal regions (PEZ_FLAIR−_) in this cohort (Fig. 5a). This observation indicates that conventional FLAIR imaging alone may fail to identify a substantial fraction of tissue at risk for recurrence. Overall, recurrent tissue represented a smaller portion of the total PEZ volume compared with non-recurrent tissue, consistent with focal patterns of tumor recurrence (Table 2).

**Table 2:** Volumes of recurrent (PEZ_T1+_) and non-recurrent (PEZ_T1−_) tissue within the *peri*-enhancing zone (PEZ). Volumes are shown as mean ± standard deviation. The last column indicates the proportion of the total PEZ volume represented by recurrent tissue (PEZ_T1+_) within each FLAIR-defined subregion.

**Region**	**PEZ_T1+_ (mm^3^)**	**PEZ_T1−_ (mm^3^)**	**% of PEZ_T1+_**
PEZ_FLAIR+_	4271 ± 4131	20162 ± 13874	17.5%
PEZ_FLAIR−_	2040 ± 2645	12859 ± 9050	13.7%

### 3.3 Diffusion characteristics (ADC)

Quantitative MRI revealed pronounced differences in tissue characteristics depending on FLAIR status. Within FLAIR-hyperintense PEZ (PEZ_FLAIR+_), recurrent regions exhibited lower ADC compared with non-recurrent tissue (1.19 ± 0.19 × 10^–3^ mm^2^/s vs. 1.29 ± 0.16 × 10^–3^ mm^2^/s; p ≈ 0.0007; Fig. 5b), reflecting higher cellularity at the invasive tumor front (Chiang, 2004, Kolakshyapati, 2018, Lee, 2011, Lu et al., 2003). In contrast, ADC differences were negligible in FLAIR-normal PEZ (PEZ_FLAIR−_: 0.99 ± 0.13 × 10^–3^ mm^2^/s vs. 0.97 ± 0.10 × 10^–3^ mm^2^/s), suggesting that baseline diffusion changes are largely confined to FLAIR-abnormal tissue. These patterns were consistent across low- and high-percentile ADC measures (Supplementary Figs. S1a–b), supporting the robustness of the findings. Measured ADC values are summarized in Table 3.

**Table 3:** Group-mean measurements of apparent diffusion coefficient (ADC) and relative cerebral blood volume (rCBV) ± standard deviation in the *peri*-enhancing zone (PEZ). PEZ_T1+_: recurrent tissue within the PEZ; PEZ_T1−_: non-recurrent tissue within the PEZ. PEZ_FLAIR+_: FLAIR-hyperintense PEZ; PEZ_FLAIR−_: FLAIR-normal PEZ. ADC values are expressed in × 10^–6^ mm^2^/s, and rCBV values are unitless. The % difference indicates the relative change of each measure in PEZ_T1+_ compared with PEZ_T1−_ within the same region. “ns” denotes a non-statistically significant difference as determined by the Wilcoxon signed-rank test.

**Region**	**Measure**	**PEZ_T1+_**	**PEZ_T1−_**	**% difference**	**p-value**
PEZ_FLAIR+_	ADC	1192 ± 188	1291 ± 162	−8.2%	0.0007
	ADC_low_	1073 ± 183	1146 ± 140	−6.3%	0.0058
	ADC_high_	1313 ± 223	1440 ± 197	−8.8%	0.0002
	rCBV	0.33 ± 0.08	0.29 ± 0.07	+13.5%	0.006
	rCBV_low_	0.25 ± 0.08	0.21 ± 0.07	+19.0%	0.005
	rCBV_high_	0.40 ± 0.09	0.36 ± 0.08	+11.1%	0.02
PEZ_FLAIR−_	ADC	987 ± 131	971 ± 102	+1.6%	ns
	ADC_low_	850 ± 108	808 ± 82	+5.2%	ns
	ADC_high_	1093 ± 158	1100 ± 137	−0.6%	ns
	rCBV	0.36 ± 0.08	0.35 ± 0.05	+2.9%	ns
	rCBV_low_	0.29 ± 0.09	0.26 ± 0.05	+11.5%	ns
	rCBV_high_	0.45 ± 0.09	0.43 ± 0.05	+4.7%	ns

### 3.4 Perfusion characteristics (rCBV)

rCBV demonstrated an opposite pattern to ADC. Within FLAIR-hyperintense PEZ (PEZ_FLAIR+_), recurrent regions exhibited higher perfusion compared with non-recurrent tissue (0.36 ± 0.08 vs. 0.33 ± 0.08; p ≈ 0.006; Fig. 5c), reflecting increased microvascular proliferation at the invasive tumor front (Jain, 2011, Vallatos, 2019, Alvarez-Torres, 2022, Hu, 2012). In contrast, rCBV differences were negligible in FLAIR-normal PEZ (Table 3, rCBV in PEZ_FLAIR−_), suggesting that baseline perfusion changes are largely confined to FLAIR-abnormal tissue. These patterns were consistent across low- and high-percentile rCBV measures (Supplementary Figs. S1c–d), supporting the robustness of the findings. Measured rCBV values are summarized in Table 3.

### 3.5 Correlation between diffusion and perfusion

ADC and rCBV were inversely correlated in PEZ_FLAIR+_ regions for both recurrent and non-recurrent tissue (r = –0.59 and –0.60, respectively; p < 0.0005; Fig. 5d), supporting a complementary relationship between tissue microstructure and perfusion in areas at risk for recurrence. No significant correlations were observed in PEZ_FLAIR−_ regions, consistent with the absence of detectable diffusion or perfusion differences in FLAIR-normal tissue.

### 3.6 Discriminatory performance

ROC analysis for group-mean PEZ_FLAIR+_ values revealed measurable discriminatory performance, with ADC and rCBV yielding AUCs of 0.69 and 0.66, respectively (Fig. 5f). Low- and high-percentile ADC and CBV metrics demonstrated similar performance (Supplementary Figs. S1e–f), confirming the robustness of these findings. In contrast, neither ADC nor rCBV provided meaningful separation between recurrent and non-recurrent tissue within PEZ_FLAIR−_ (Fig. 5b–c), highlighting the current limitations of standard quantitative MRI in FLAIR-normal regions.

## 4 Discussion

We present a three-dimensional (3D) volumetric evaluation of preoperative ADC and CBV within the *peri*-enhancing zone (PEZ) of glioblastoma. Regions within FLAIR-hyperintense PEZ (PEZ_FLAIR+_) that later developed recurrence (PEZ_T1+_) exhibited significantly lower ADC and higher rCBV than recurrence-free tissue (PEZ_T1-_). Notably, approximately one-third of recurrent tissue was located in baseline FLAIR-normal PEZ (PEZ_FLAIR-_), where neither ADC nor rCBV detected differences.

The PEZ is a critical target for surgical resection and radiotherapy (Lemée et al., 2015, Giambra, 2023), yet preoperative characterization remains challenging (Martin-Noguerol et al., 2021, Michalska-Foryszewska, 2024). Our study focused on a 5-voxel-wide margin surrounding the contrast-enhancing tumor core, capturing tissue at highest risk for recurrence. Abnormal PEZ regions at diagnosis (PEZ_T1+_) were defined based on follow-up T1c enhancement mapped to baseline MRI, including necrotic areas and residual resection cavity, ensuring that regions beyond the contrast-enhancing tumor removed during surgery were included. In contrast, normal PEZ regions (PEZ_T1-_) were defined as areas without T1c enhancement across both imaging time points. FLAIR hyperintensity in the PEZ reflects biologically distinct tissue, including edema and infiltrative tumor cells, which differ from FLAIR-normal regions in cellularity and microenvironment (Giambra, 2023). Accordingly, separate analysis of PEZ_FLAIR+_ and PEZ_FLAIR−_ regions enables biologically and clinically meaningful comparisons, allowing quantitative MRI markers to be interpreted in the context of these distinct tissue compartments. The proposed 3D image preprocessing and analysis framework facilitates quantitative evaluation of voxel-wise tissue changes by linking baseline and follow-up MRI.

ADC has been associated with tumor cellularity and patient survival, with lower ADC in *peri*-enhancing regions linked to future recurrence (Baehring and Fulbright, 2012, Gaddamanugu, 2022, Pope, 2011, Pope, 2012, Saksena, 2010, Wen, 2015). Several studies showed that ADC values in *peri*-enhancing regions are indicative of neoplastic cell infiltration (Chiang, 2004, Kolakshyapati, 2018, Lee, 2011, Lu et al., 2003). Elson et al. (Elson, 2015) found that recurrence overlapped ∼ 60% with diffusion-restricted PEZ regions in 88% of cases, correlating with progression-free survival. Histogram-based analyses of recurrent glioblastoma similarly linked lower mean ADC to poorer outcomes (Pope, 2009), and multiple studies observed reduced ADC in regions surrounding the gross tumor volume that subsequently recurred (Chang et al., 2017, Jeong, 2015, Matsuda et al., 2022, Xing, 2024).

CBV can help to distinguish pseudoprogression and radiation necrosis from tumor progression and predicts treatment response in gliomas (Barajas, 2009, Bennett, 2017, Blasel, 2016, Choi et al., 2013, Khalifa, 2016, Kickingereder, 2016, Nael, 2018, Sawlani, 2010, Singh, 2016). CBV metrics also associate with clinical outcomes: higher CBV predicts shorter time to progression and worse prognosis (Boxerman et al., 2006, Jain, 2014), while texture features in both the tumor core and *peri*-enhancing regions predict overall survival (Lee, 2016). Most relevant to our work, Xing et al. (Xing, 2024) found higher preoperative CBV in *peri*-enhancing regions that later developed recurrence.

In this cohort, lower ADC within recurrent PEZ_FLAIR+_ regions aligns with prior reports linking diffusion restriction to increased tumor cellularity (Baehring and Fulbright, 2012, Gaddamanugu, 2022, Kono, 2001) and future recurrence (Chang et al., 2017, Jeong, 2015, Matsuda et al., 2022, Xing, 2024), while elevated CBV reflects microvascular proliferation and infiltration (Jain, 2011, Vallatos, 2019). We found only one study, which evaluated ADC in conjunction with CBV within the *peri*-enhancing zone (Xing, 2024). They reported similar patterns, showing lower ADC and higher CBV in *peri*-enhancing tissue destined for recurrence (Xing, 2024). The observed correlation between ADC and CBV further supports a complementary relationship between tissue microstructure and perfusion in regions predisposed to recurrence. Our findings highlighting the value of integrating these qMRI metrics for recurrence assessment.

ROC analysis demonstrated modest discriminatory performance, with ADC and rCBV yielding AUCs of 0.69 and 0.66, respectively. Although limited for direct clinical application, this accuracy is reasonable given that it was based on single mean values of the 3D volumes, which inherently obscure fine voxel-level differences. Nevertheless, even this coarse-level approach supports the feasibility of detecting local tissue alterations associated with recurrence using qMRI. We speculate that more sophisticated approaches, such as deep-learning models integrating multiparametric qMRI features with Positron Emission Tomography (PET-MRI), could achieve clinically relevant accuracy.

Our findings demonstrate that baseline quantitative MRI contains measurable information within the *peri*-enhancing zone that is not captured by conventional visual assessment alone. Specifically, lower ADC and higher CBV within FLAIR-hyperintense PEZ regions that later recur suggest increased cellularity and microvascular proliferation at the invasive tumor front, consistent with known histopathological features of glioblastoma infiltration (Lemée et al., 2015, Giambra, 2023, Hara, 2019). Importantly, these differences were detectable preoperatively using routinely acquired diffusion and perfusion MRI, highlighting the potential of qMRI to refine risk stratification beyond standard anatomical imaging. At the same time, the absence of detectable ADC or CBV differences in FLAIR-normal PEZ—despite accounting for a substantial proportion of recurrent tissue—underscores a critical limitation of current imaging approaches and reinforces the need for complementary modalities or advanced quantitative techniques. Together, these findings support the PEZ as a biologically and clinically meaningful target for imaging-based assessment of recurrence risk and emphasize the value of three-dimensional, spatially resolved analysis for neuroradiological evaluation and treatment planning.

According to the RANO (Wen, 2023) initiative, the standard glioblastoma MRI protocol mandates diffusion-weighted MRI (ADC) for quantitative assessment, while perfusion MRI (CBV) is optional but recommended. Other quantitative sequences are not required in routine imaging. In our study, ADC and CBV were informative but insufficient to fully capture the biological heterogeneity of the *peri*-enhancing zone. We speculate that applying deep learning or other predictive models using only the standard clinical MRI protocol may be insufficient to achieve reliable recurrence prediction, which is consistent with the fact that no such model has yet been adopted clinically. We emphasize that this does not reflect limitations of the published methods themselves, but rather the restricted input data currently available. While ADC and CBV demonstrated promising discriminative ability, a substantial fraction of recurrent tissue remained indistinguishable from stable tissue at baseline. These findings highlight both the potential and the current limitations of ADC and CBV and suggest that the standard glioblastoma imaging protocol by RANO could benefit from revision toward a more quantitative and computationally oriented framework. Incorporating additional qMRI sequences—such as dynamic contrast-enhanced MRI (Heye et al., 2014), T1 and T2 relaxometry (Chekhonin, 2024), quantitative susceptibility mapping (Liu, 2015), BOLD (Abu Mhanna, 2025) imaging, or intravoxel incoherent motion diffusion (Mesny et al., 2024)—may enable more comprehensive tissue characterization and enhance the predictive value of ADC and CBV for glioblastoma recurrence. Further research is needed to identify the most informative sequences for a standardized clinical protocol, and the 3D framework presented here provides a robust methodological foundation for such investigations.

Methodological considerations include the use of absolute ADC and rCBV values normalized to the cohort mean histogram, rather than relative measures against reference tissue. This approach is justified given the uniform acquisition protocol, paired within-patient comparisons, and single-time-point analysis. Prior work demonstrates that absolute qMRI metrics provide biologically meaningful measures of tissue cellularity and vascularity, avoiding confounds from interhemispheric differences or ROI placement variability (Hoxworth, 2020, Qin, 2018). ADC and rCBV maps were normalized using a histogram-based scaling procedure to reduce inter-subject variability while preserving biologically relevant contrast. Given the homogeneous acquisition protocol and the paired within-patient analytical framework, this standard normalization approach provides robust and meaningful quantitative measures. The effect of more complex normalization methods (e.g., alpha-stable distribution–based approaches), while potentially beneficial in multi-scanner or heterogeneous datasets, is expected to be minimal on the final results in the present study.

Limitations of this study include its retrospective, single-center design, and the focus on local recurrence. The presented framework relies on a seed volume for the generation of the *peri*-enhancing zone, which is the baseline tumor core. Therefore, extension to nonlocal recurrence will require methodological adaptations. Direct correlation with histopathological or molecular features was not feasible, limiting interpretation of tissue composition underlying qMRI alterations. In addition, image registration between baseline and follow-up MRI is inherently challenging due to the significant mass effect of glioblastoma and brain shift; this difficulty has even prompted dedicated machine learning challenges. In our cohort, visual inspection indicated that only one patient needed to be excluded due to poor registration. Moreover, because our analysis focused on the relatively thin 5-voxel-wide PEZ, registration errors within this region are expected to be minimal and further mitigated by the use of mean value statistic. Nevertheless, the results highlight the potential of 3D quantitative imaging to capture spatial heterogeneity and guide future investigations integrating imaging and histology.

## 5 Conclusion

Overall, this study provides both a methodological and conceptual framework for quantitative, region-of-interest–based analysis of the *peri*-enhancing zone (PEZ) in glioblastoma integrating baseline and follow-up MRI and emphasizes the importance of this region from a neuroradiological perspective. Within FLAIR-hyperintense PEZ regions, recurrent tissue exhibited lower ADC and higher CBV compared with stable tissue, whereas no detectable differences were observed in FLAIR-normal PEZ despite representing a substantial portion of recurrent volume. These findings demonstrate that baseline quantitative MRI contains clinically and biologically relevant information beyond conventional anatomical imaging, supporting the PEZ as a meaningful target for imaging-based tumor characterization. Importantly, the absence of detectable differences in a substantial fraction of recurrent tissue underscores the limitations of currently available routine quantitative MRI markers and highlights the need to incorporate additional quantitative MRI parameters into standard glioblastoma imaging protocols.

## CRediT authorship contribution statement

**Gergely Bertalan:** Writing – review & editing, Writing – original draft, Visualization, Software, Methodology, Investigation, Formal analysis, Data curation, Conceptualization. **Nicolin Hainc:** Writing – review & editing, Writing – original draft, Visualization, Validation, Methodology, Investigation, Formal analysis, Data curation, Conceptualization. **Gaetan Paignon:** Visualization, Formal analysis, Data curation. **Ramona A. Todea:** Writing – review & editing, Writing – original draft, Validation, Methodology, Data curation, Conceptualization. **Andrea Bink:** Writing – review & editing, Writing – original draft, Validation, Methodology, Data curation, Conceptualization. **Tobias Weiss:** Writing – review & editing, Writing – original draft, Data curation. **Michael Weller:** Writing – review & editing, Writing – original draft, Methodology, Data curation, Conceptualization. **Carlo Serra:** Writing – review & editing, Writing – original draft, Conceptualization. **Zsolt Kulcsar:** Writing – review & editing, Writing – original draft, Validation, Supervision, Project administration, Methodology, Funding acquisition, Conceptualization.

## Funding

The authors did not have a specific grant for this research from any funding agency in the public, commercial or not-for- profit sectors.

## Declaration of competing interest

The authors declare that they have no known competing financial interests or personal relationships that could have appeared to influence the work reported in this paper.

## Data Availability

Data will be made available on request.
