# Supplementary material for: Three-Dimensional quantitative analysis of the Peri-Enhancing zone reveals ADC and CBV signatures of glioblastoma recurrence
Source: Neuroimage Clin. 2026 Mar 4;49:103977. doi: 10.1016/j.nicl.2026.103977 (PMC12993171; doi:10.1016/j.nicl.2026.103977)
Supplement: Supplementary Data 1 [file mmc1.docx]

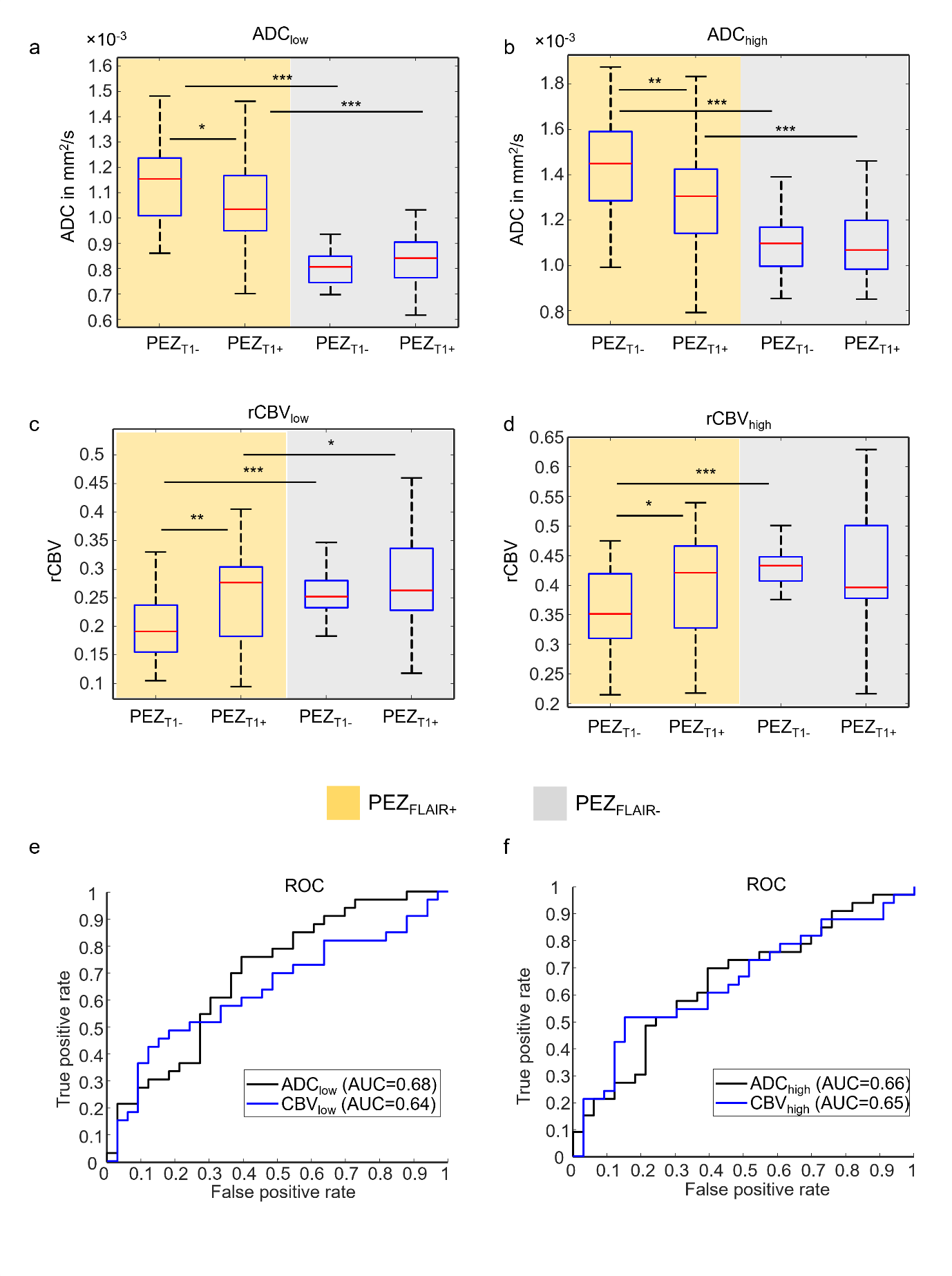


**Supplementary Figure S1:** Subgroup analyses of ADC and rCBV in the peri-enhancing zone (PEZ). (a–b) ADC analyses for low- and high-value subgroups: (a) excluding the top 20^th^ percentile of ADC values, and (b) excluding the bottom 20^th^ percentile. (c–d) rCBV analyses for low- and high-value subgroups: (c) excluding the top 20^th^ percentile of rCBV values, and (d) excluding the bottom 20^th^ percentile. Analyses were performed analogous to Fig. 5b–c. Significance levels from Wilcoxon signed-rank tests are indicated as follows: *p < 0.05, **p < 0.005, ***p < 0.0005. (e-f) ROC–AUC analysis of low- and high-value ADC/rCBV subgroups.
